# Traditional Chinese Medicine and regulatory roles on epithelial–mesenchymal transitions

**DOI:** 10.1186/s13020-019-0257-6

**Published:** 2019-09-23

**Authors:** Jing Bai, Wee Chiew Kwok, Jean-Paul Thiery

**Affiliations:** 1 Department of Mechanical Engineering, Massachusetts Institute of Technology, Cambridge, USA; 2 Department of Biochemistry, Yong Loo Lin School of Medicine, National University of Singapore, Singapore, Singapore; 3 Guangzhou Regenerative Medicine and Health, Guangdong Laboratory, Guangzhou, China

**Keywords:** Epithelial–mesenchymal transition, Traditional Chinese Medicine, Fibrosis, Wound-repair, Cancer invasion and metastasis, Stromal cells

## Abstract

Epithelial–mesenchymal transition (EMT) is a critical biological process allowing epithelial cells to de-differentiate into mesenchymal cells. Orchestrated signaling pathways cooperatively induce EMT and effect physiological, sometimes pathological outcomes. Traditional Chinese Medicine (TCM) has been clinically prescribed for thousands of years and recent studies have found that TCM therapies can participate in EMT regulation. In this review, the historical discovery of EMT will be introduced, followed by a brief overview of its major roles in development and diseases. The second section will focus on EMT in organ fibrosis and tissue regeneration. The third section discusses EMT-induced cancer metastasis, and details how EMT contribute to distant dissemination. Finally, new EMT players are described, namely microRNA, epigenetic modifications, and alternative splicing. TCM drugs that affect EMT proven through an evidence-based research approach will be presented in each section.

## Background

Epithelial–mesenchymal transition (EMT) is the dynamic progression, from well-differentiated epithelial cells into partial or complete fibroblastoid-like mesenchymal cells. Distinct epithelial characteristics are progressively lost, from a specialized apicobasal polarity to a front-rear polarity. Epithelial cell–cell adhesions also dissociate to enable migratory freedom. This reversible EMT process is vital and highly regulated in different stages of embryonic development. Prior to the understanding of EMT, 19th century embryologists had long observed mesenchymal phenotype within epithelial structures of developing tissues. The Matthias Duval’s 1879 hand-drawn atlas of the chicken embryo documented a distinct neural-crest cell population alongside epithelial and mesenchymal cell phenotypes. Ramon y Cajal suggested in 1890 that breast carcinoma cells can delaminate from a cell cluster to invade the adjacent stroma. The concept of EMT was eventually established in the 1960s by Elizabeth Hay through numerous morphological studies conducted on chicken embryos [1–3]. The study on EMT in vivo was challenging but Hay and Greenburg inadvertently developed an in vitro model in 1982 to study EMT. Using cells from the adult anterior lens and various embryonic tissues, they observed that epithelial cells undergo major organizational changes when plated on three-dimensional (3D) collagen gels [4]. The environmental conditions generated migratory cells with shape and polarity indistinguishable from mesenchymal cells. This 3D in vitro model allowed further experimentation of EMT processes and the development of different extracellular matrix and diffusible factors [5–8]. Delving deeper into the underlying mechanisms, Sir Michael Stoker discovered in 1987 that fibroblast-derived scatter factor induces Madin–Darby canine kidney (MDCK) epithelial cell dispersion through a paracrine signaling mechanism [9].

Developmental biologists uncovered multiple signaling pathways controlling EMT during morphogenesis and organogenesis. The fibroblast growth factor (FGF), wingless-type (Wnt), and transforming growth factor-beta (TGF-β) signaling pathways crosstalk at different levels to coordinate development [10–12]. The scatter factor discovered by Michael Stoker was eventually identified as the hepatocyte growth factor (HGF) [9, 13, 14]. Together with other growth factors such as FGF-1 and TGFβ [15, 16], normal or transformed epithelial cells were encouraged to disperse. However, extracellular matrix molecules [17] growth factors and extracellular molecules were essential to first stimulate EMT in epithelial cells [18–20]. In 1994, the zinc-finger protein SLUG [21] was shown to control EMT in chicken embryo gastrulation. An orthologous master regulatory transcription factor, *Snail*, was found to mediate mouse gastrulation in a similar fashion. The two related transcription factors, now designated *Snai1* (Snail) and *Snai2* (Slug), are involved in neural crest ontogeny of different vertebrate embryos [22]. Interestingly, even in invertebrates such as the drosophila, the ortholog of *Snai1* designated *Snail* contributes to cell shape changes in the formation of the ventral furrow during gastrulation [23]. All these findings support the notion that EMT is a universal process that is conserved during evolution.

**Fig. 1:**
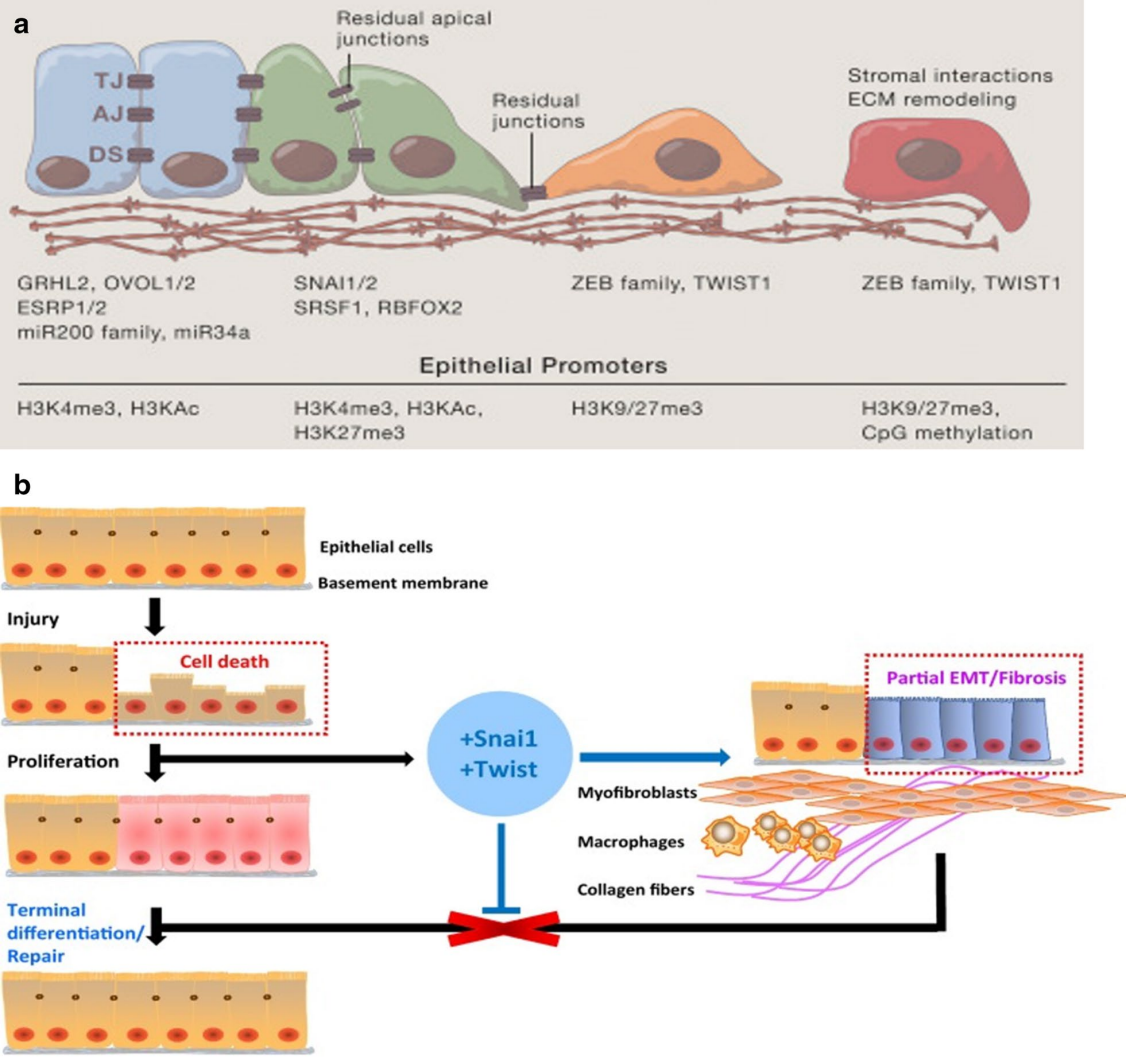
EMT and organ fibrosis. **a** EMT can be considered as a continued spectrum, where cells present epithelial (E), intermediate (EM), and mesenchymal (M) phenotypes. During EMT, epithelial cells sequentially lose apico-basal polarity and cell–cell adhesions gain front-back polarity and enhanced cell–matrix interactions [27]. **b** Cell death induces regenerative process, e.g. epithelial cell proliferation. Upregulation of Snai1 and Twist inhibits EC differentiation, leads to myofibroblast and inflammatory cell accumulation and fibrosis [132] (permission obtained from original publishers.)

Unfortunately, the role of EMT extends beyond normal development to disease progression such as tumorigenesis and fibrosis. Discovery of *SNAI2*-induced EMT in bladder carcinoma cell line [24] reaffirms that EMT promote metastasis in carcinoma through dissolution of desmosomes and remodeling of cytoskeletal proteins. Studies suggest that EMT regulates organ fibrosis [25–27] for instance, through inducing cell cycle arrest and parenchymal damage in renal fibrosis [28]. Figure 1a depicts the EMT process.

The unwanted implications of EMT in pathogenesis is a motivating factor for scientists to harness viable alternative medicine. Evolved over thousands of years, Traditional Chinese Medicine (TCM) is a medical practice that considers disease as a result of imbalances in the macro-environment. Instead of targeting the local symptoms, treatments are based on a system-wide, multi-target, multi-level holistic approach. Coincidentally, to mitigate the risk of drug resistance, modern medicine is embracing the concept of multi-target therapeutics. The integrative approach in TCM diagnosis and treatment has shown promising effect on EMT in tissue remodeling to cancer progression. With the potential of TCM treatment recognized, majority of cancer patients in China have received TCM in their cancer treatment [29]. Modern drug delivery systems such as targeted drug delivery system (TDDS) also accelerate the applications of TCM on cancer therapy. Passive-targeting TCM preparation, liposomes, emulsion and microspheres offer enormous advantages on traditional drug delivery methods of oral absorption, injection or topical administration [30, 31], allowing for drugs to be concentrated on the target site with higher efficacy and lower side-effects. In this review, we will explore the role of TCM in EMT intervention and discuss novel therapeutic targets for EMT-related organ fibrosis and tumorigenesis.

## Tissue remodeling

### Organ fibrosis

Organ fibrosis is initiated by a repetitive or persistent injury which in turn trigger inflammatory immune cells to produce cytokines and subsequently recruit and activate quiescent interstitial fibroblasts. Activated contractile fibroblasts, called myofibroblasts, produce excessive extracellular matrix components that deposit as a fibrous network on existing tissue architecture, ultimately augmenting and impairing organ function. Besides arising from chronic injury, myofibroblasts are believed to originate in part from epithelial cells via EMT [32]. In vivo experiments on mouse kidney fibrosis showed that about 30% of fibroblasts are derived from EMT-induced epithelial cells [33]. Studies on fibrotic tissues of the lung, liver, heart and lens also documented EMT as an important source of fibroblasts, as well as perivascular smooth muscle cells and pericytes [34]. Endothelial cells which are progenitor cells of smooth muscle cells and pericytes, are proposed to transform into mesenchymal cells via endothelial–mesenchymal transition (EndMT) [35, 36]. EndMT and EMT are complex biological processes generating mesenchymal-like cells. The cell of origin differs in these two processes, EMT occurs in epithelial cells derived from ectoderm or endoderm. EndEMT occurs in endothelial cells deriving from mesoderm [37]. Nonetheless, EndMT and EMT process activate common transcription factors, such as Snail, Slug, Twist, ZEB1, ZEB2, and Sox2. However, downstream signaling for EndMT results in repressing the expression of endothelial genes (e.g. CDH5 and PECAM1) and subsequently activating the expression of mesenchymal genes (e.g. VIM and COL5A1) [38]. TGF-β1, commonly associated with EMT, is also a major regulator of EndMT in capillary endothelial cells by reducing endothelial markers CD31 and integrin αVβ3 while expressing FSP1, α-SMA, DDR2, collagen I, and vimentin [34]. β-catenin, a central mediator of the Wnt pathway, is an integral member of cell–cell adherens junctions and together with TGF-β1 are common markers used to study the mechanisms in EndMT [39–42]. Grande et al. studied the mechanisms in detail using a renal fibrosis model and observed that the epithelium does not contribute directly to the increased pool of myofibroblasts [28], Instead, epithelial cells undergo partial EMT, losing epithelial specific markers and its apico-basal polarity while remaining integrated in the tubules [26]. They also produce various cytokines and exosomes that recruit bone marrow mesenchymal progenitors and macrophages exacerbating the fibrotic response [27]. Figure 1b depicts the EMT and organ fibrosis.

A complex molecular and cellular network orchestrates pathological EMT during fibrosis including TGF-β, EGF and FGF2. Using the CCl_4_-induced liver fibrosis model, the potential of a multi-component traditional herbal formulae, Han-Dan-Gan-Le, as a prescribed treatment was investigated. The rats administered with Han-Dan-Gan-Le compared to the control group displayed 50% lower CCl_4_-induced hepatic collagen accumulation [43]. Diwu Yanggan (DWYG), which consists of five Chinese herbs, provoked the reversal of EMT to mesenchymal-to-epithelial transition (MET) in the fibrotic liver tissues, with the up-regulation expression of E-cadherin and down-regulation expression of vimentin [44]. The main constituent of DWYG, curcumin, was found in other studies to inhibit TGF-β-Induced EMT via PPARγ pathway in renal tubular epithelial cells in tubulointerstitial fibrosis [45]. Another example is provided by the Wenyang Huazhuo Tongluo formula, which inhibits fibrosis via suppressing Wnt/β-catenin signaling pathway [46]; and combination of *Salvia miltiorrhiza* and ligustrazine attenuates bleomycin-induced pulmonary fibrosis in rats via modulating TNF-α and TGF-β [47]. The multi-component, multi-level and multi-targeted properties of TCM treatment limit complete understanding of its mechanisms but it holds great clinical potential in the treatment of fibrosis as demonstrated in the examples above. Table 1 provides a list of TCM herbs and prescriptions regulating pathological EMT.

**Table 1:** Examples of TCM regulating pathological EMT [19, 133]

Wenyang Huazhuo Tongluo formula	Wnt/β-catenin	Skin fibrosis [46]
Salvia miltiorrhiza and ligustrazine	TNF-α and TGF-β	Pulmonary fibrosis [47]
Genistein	IL-6, TNFα, myeloperoxidase SFRP1 and SMAD4	Hepatic fibrosis [18] Prostate cancer [127]
Betulin	TNF-α, TGF-β1, TIMP-1, TIMP-2/MMP-2	Liver fibrosis [19]
Silybinin	TGF-β1/oxidase stress	Liver fibrosis [133]
Diwu Yanggan formula	Modulated the EMT and MET balance	Liver fibrosis [44]
Salvianolic acid B	Abrogated EMT-induced fibrogenesis	Renal fibrosis [134]
Fuzheng Huayutablet	α-SMA, TGF-β1, and SMAD3	Renal fibrosis [135]
Handan Ganle	Modulated the EMT and MET balance	Liver fibrosis [136]
Shikonin	Stimulate EMT related microRNAs	Skin wound healing [55]
Tanshinone IIA	TLR4/MYD88/NF-κB	Ventricular remodeling [56]
Curcumin	TGF‐β/SMAD and SNAIL; PI3 K/AKT/mTOR; PPARγ	Renal fibrosis [45] /hepatocellular [89] /lung carcinoma [137]
Polyphyllin I	IL-6/STAT3	Non-small-cell lung cancer [138]
Ginsenoside Rg3	NF-κB	Lung cancer [69]
Matrine	ROS/NF-κB/MMPs	Pancreatic cancer [117]
Toosendanin	AKT/mTOR	Pancreatic cancer [139]
Brucine	MMP-2 and MMP-9	Breast cancer [140]
Yiqi Huayu Jiedu decoction	TGF‐β/SMAD	Gastric cancer [141]
Yi Ai Fang	HIF‐1α	Colorectal cancer [116]
Jianpi Huayu decoction	SMAD3/SMAD7	Hepatocellular carcinoma [90]
Resveratrol	LPS‑induced EMT	Prostate cancer [70]
Triptolide	MMP‐7 and MMP‐19	Ovarian cancer [51]
Thymoquinone	TWIST1/ZEB1/E-cadherin	Cervical cancer [72]
Shaoyao decoction	IL1β, IL6, TNF-α, tumor-associated macrophages, and p65	Colorectal cancer [94]
Qingyihuaji formula	Tumor-associated macrophage and IL-6	Pancreatic cancer [67]
Sheng Qi formula	Myeloid immune-suppressor cells (MDSC)	Breast cancer [81]
Feiyanning decoction	CD4+ CD25+ regulatory T cells	Lewis lung carcinoma [80]
Ginsenoside Rb1	MicroRNA (mir)-25	Ovarian cancer [66]
Jin-Fu-An decoction	P120ctn, isoform 1A	Lung cancer [129]

### Tissue regeneration

Upon injury, inflammatory signaling modulators recruit diverse types of cells, most predominantly macrophages. These professional phagocytic cells can trigger EMT through the production of growth factors, such as TGF-β, PDGF, EGF, and FGF-2 [48–50]. In addition, these cells also secrete chemokines and metalloproteases (MMPs), notably MMP-2, MMP-3, and MMP-9 [51]. During skin wound-healing, keratinocytes undergo a partial EMT process. These keratinocytes, together with surrounding cells induce basement membrane damage while sustaining an intermediate phenotype through the transient expression of *SNAI2* [52]. The recruitment of keratinocytes in the wounded area can be inhibited by blocking MMP activity [53]. EGF signaling is also demonstrated to activate EMT in ovarian surface epithelial cells in each menstrual cycle, via MMPs and ILK/ERK kinase [54]. Subsequent phases of wound-healing involve collagen deposition, granulation tissue formation followed by re-epithelialization. These processes may utilize EMT and MET in an orchestrated manner and mediate cell proliferation while restoring the epithelium integrity. Shikonin, a major component of zicao, a TCM herb, has been reported to stimulate EMT in skin wound-healing [55]. Tanshinone IIA [56] another TCM herb, reduces macrophage infiltration and inflammation by inhibiting the TLR4/MyD88/NF-kB pathway. These findings shed new light on developing therapeutic strategies for tissue regeneration.

## Cancer progression

### Dynamics of junctional complexes in the progression of carcinoma

Carcinoma, which derive from the malignant transformation of epithelial cells, account for 90% of all cancers. Progression of in situ carcinoma to the invasive stage is lethal since carcinoma cells can disseminate and affect distant organs. EMT equip carcinoma with invasive properties capable of disseminating into surrounding stroma and is hypothesized to be the main contributor of metastatic shift in carcinoma.

Epithelial-specific adhesion structures: tight junctions (TJs), adherens junctions (AJs) and desmosomes [57, 58] are dysregulated during EMT. Selectively permeable, TJs allow trans-epithelial transport of ions but forms a physical barrier to fluid movement between the lumen and the stroma. TJs can be destabilized by TGF-β signaling, leading to RhoA degradation and disruption of actin microfilaments [59, 60]. Adherens junctions localized below the tight junctions in polarized epithelial is the first adhesive structure to form following contact between epithelial cells. The junctions initially appear as E-cadherin-containing punctates, which can ultimately assemble into a cortical belt associated with a unique cortical actin microfilament. The E-cadherin cytoplasmic domain controls the dynamics of actin microfilaments assembly and the decrease or disappearance of this prominent EMT marker is a critical step in EMT regulation [61]. The down-regulation of E-cadherin through epigenetic repression is mediated by transcription factors from the Snail, Zeb and Twist families effecting on CpG islands in the promoter region or histone methyltransferases on chromatin organization [62]. E-cadherin can also be down-regulated by post-translational modifications. HGF, EGF and FGF-activating receptor tyrosine kinases (RTK), induce EMT together with the phosphorylation and subsequent ubiquitination and degradation of E-cadherin [63, 64]. Desmosome adhesion is achieved via desmocollins and desmogleins, members of the calcium-dependent cadherin superfamily. The cytoplasmic domain of the desmosomal cadherins binds to plakoglobin, a member of the catenin family, and to desmoplakin which is connected to cytokeratin intermediate filaments [65]. Serine phosphorylation by members of the protein kinase C (PKC) family helps to maintain desmosome stability. In a laboratory setting, monolayer confluency confers desmosome with a calcium-independent property, and thus, stability even from divalent cation disruption [66].

Well-developed TJs are absent in the early intermediate stage of EMT while AJ destabilization, usually accompanied by desmosome disassembly, is the hallmark of the intermediate late stage of EMT. Initially considered as an epithelial to mesenchymal transformation process, many EMT-induced carcinomas remain in intermediate states during EMT. Intermediaries oscillate in their phenotypes and possess relative ability to reassemble immature AJ. Expression of major AJ constituent, E-cadherin, is repressed by *ZEB1*, a transcription factor regulated by miR200 family members [67]. The layered feedback loops form a regulatory cascade that affect E-cadherin turnover and subsequently, EMT progression [29]. To reach complete mesenchymal state will require tight control of adhesive components by transcriptional repressors and epigenetic marks [62, 68].

The effect of TCM herbs on EMT is extensive and not well-elucidated but some have been studied in greater detail, especially its effect on EMT-associated adhesion complex. Ginsenoside Rg3, a compound abundant in *Ginseng panax*, could significantly alter EMT markers by increasing E-cadherin and decreasing expression of *SNAI1*, N-cadherin and vimentin [69]. Resveratrol, a natural polyphenolic compound found in *Polygonum cuspidatum*, grapes and peanuts, can inhibit LPS-induced EMT morphological changes in PC-3 and LNCaP PCa prostate cancer cells [70]. LPS-exposure upregulated transcription factor glioma-associated oncogene homolog 1 (Gli1) but resveratrol inhibited this gene. In another study, resveratrol reduced human lung carcinoma cell line A549 spheroid dispersion by 40% compared to the control sample [71]. Triptolide, a compound extracted from the TCM *Tripterygium wilfordii*, inhibited metastasis of ovarian cancer cells SKOV3 and A2780 via *MMP7* and *MMP19* repression and E-cadherin activation [51]. The in vivo mouse model conducted in the same study supported the observation that triptolide can inhibit tumor formation and metastasis in nude mice. Thymoquinone, extracted from *Nigella sativa*, inhibit EMT in cervical cancer cell lines SiHa and CaSki by inactivating EMT signaling, *TWIST1* and *ZEB1* repression, and preventing cellular migration through E-cadherin activation [72].

### EMT signaling in the context of the tumor microenvironment

**Fig. 2:**
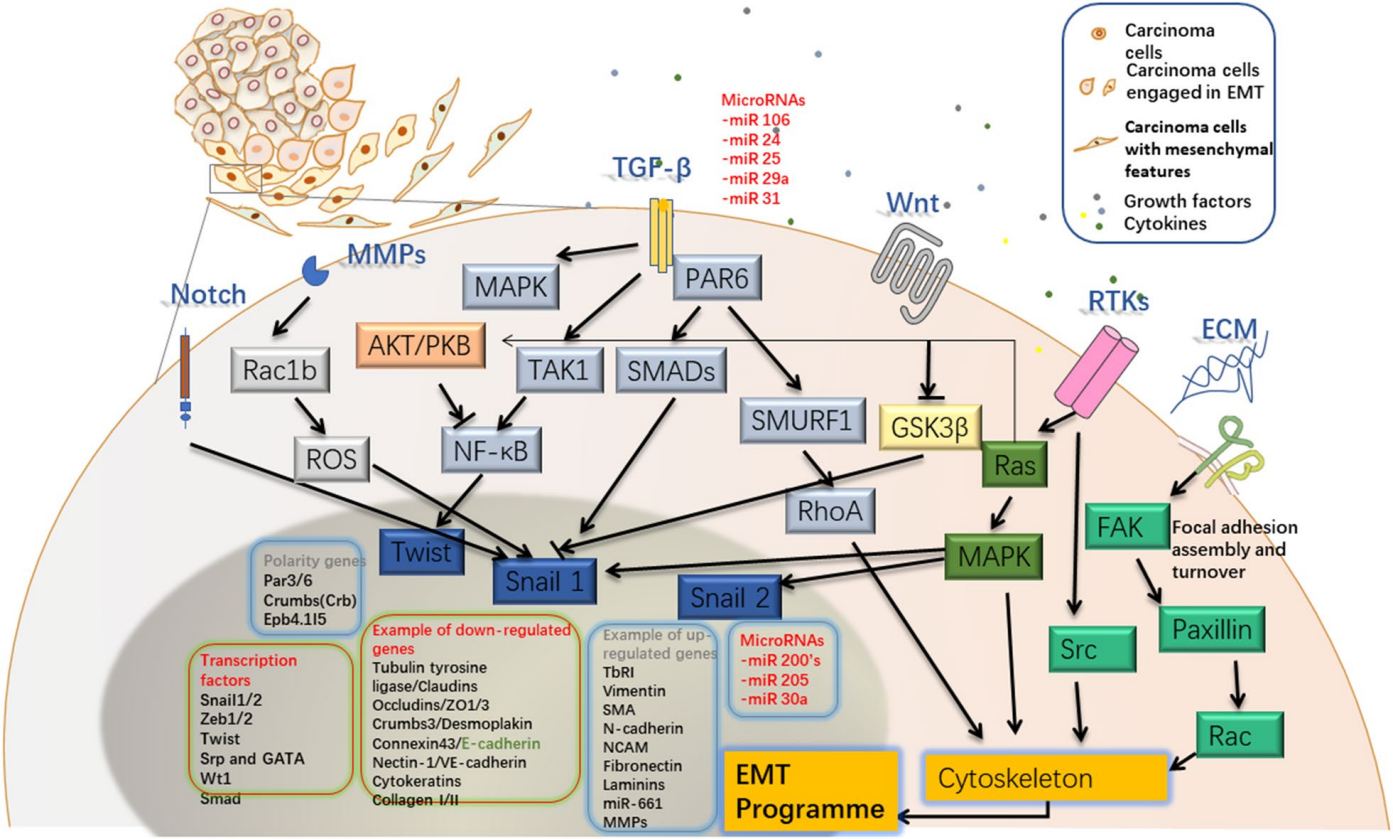
Snapshot of EMT signaling pathways. EMT in carcinoma is induced by various pathways, either by growth factors and cytokines from adjacent cells, or via cell interactions with extracellular matrix (ECM). In the paracrine mode, Receptor Tyrosine kinases (RTKs), TGFβ, Wnt and Notch can be activated. EMT could also be induced through receptors such as integrins interacting with ECM proteins like collagens, subsequently activating intracellular kinases such as Src. Signal transduction involves transcription factors and polarity genes [11, 61, 82, 88]

The tumor microenvironment is considered crucial in the initiation and progression of malignant cells [73]. Recent findings support the notion that cancer cells require support from stromal cells before it can expand autonomously. An EMT fate is dependent on the reciprocal interactions between carcinoma cells and the stroma with the interplay of growth factors, cytokines and chemokines in the microenvironment (Fig. 2). HGF, EGF, FGF, IGF, PDGF, TGF-β, Wnt family members, Sonic Hedgehog, Notch, interleukins and chemokines are signaling molecules that have been shown to induce EMT [74, 75]. EMT signaling molecules can activate diverse pathways such as Ras, Smad and β-catenin. Downstream transcription factors such as *SNAI1*, *SNAI2*, zinc finger E-box binding homeobox 1 and 2 (*ZEB1* and *ZEB2*), *TWIST1*, Forkhead boxC2 (*FOXC2*), high mobility group A2 (*HMGA2*) and paired related homeobox 1 (*PRX1*) are subsequently activated in carcinoma [62, 76–81].

TGF-β is one of the key element that promotes invasion and metastasis by altering apico-basolateral polarity, generating tumor initiating cells, and sheltering the tumor from immune surveillance and apoptosis signals [82–84]. Initiated by ligand binding to type II TGF-β receptors, the canonical TGF-β signaling leads to phosphorylation of regulatory SMAD2 and SMAD3. The non-canonical TGF-β pathway involves several non-receptor protein tyrosine kinases (SRC/FAK), p38 MAPK and the GTPase RhoA [85–87]. Curcumin inhibits TGF-β1-induced EMT in hepatoma cells by down-regulating downstream SMAD2 phosphorylation. Assembly of phosphorylated SMAD2:SMAD3 with SMAD4 into a heterotrimeric complex [88] and translocation into the nucleus to control gene transcription is prevented, effectively inhibiting EMT [89]. Jianpi Huayu Decoction (JHD), a TCM prescription against cancer, is found to halt EMT hepatocellular carcinoma by regulating the Smad3/Smad7 cascade. Smad7 expression is increased while expression of p-Smad3 and Snail decreased leading to E-cadherin up-regulation and EMT inhibition [90].

### EMT and cancer niche

An inflammatory site is often established prior to the appearance of in situ cancer cells and is termed the cancer niche [91]. The normal epithelium cells in this niche are more susceptible to stress triggers, such as chemical carcinogenesis, to undergo mutation and malignancy changes. The recruitment of bone marrow immature myeloid cells usually accompanies the onset of inflammatory stroma expansion adjacent to the dysplastic lesion. Different stromal cell types, fibroblasts, endothelial cells and pericytes, have been identified in tumours associated with angiogenesis, myeloid and lymphoid lineages [71, 92]. The stroma cells interact closely with carcinoma cells to produce many growth factors and cytokines that contribute to the acquisition of a mesenchymal-like phenotype. Although the regulation of adhesion mechanisms is crucial in the control of EMT status in carcinoma, stroma cells operate in a tissue-specific manner that affect EMT outcome. A detailed study was performed to characterize mesenchymal stems cells under ex vivo autocrine and paracrine loops from colon carcinoma cells [93]. The study found that the colon carcinoma line secreted IL-1 which induced prostaglandinE2 (PEG2) production through the cyclooxygenase2 (COX2) and microsomal prostaglandinE2 synthase-1 increased level. Secreted PEG2 promotes the autocrine production of IL-6, IL-8 and Gro-α. In addition, PEG2 induces nuclear translocation of β-catenin through inactivation of GSK3β by AKT activation. The crosstalk between colon carcinoma cells and mesenchymal stem cell led to the emergence of ALDH-1/CD133 double positive tumor initiating cells.

Shaoyao decoction (SYD), a TCM prescription that originated in the Jin-Yuan Dynasty, was found to suppress colitis-associated colorectal cancer. The inflammatory niche in colitis is addressed as SYD repressed proinflammatory cytokines IL-1β, IL-6, and TNF-α. Thus, inhibiting Snail-induced EMT by attenuating TAM infiltration and NF-κB activation [94].

EMT may prevent senescence induced by oncogenes, therefore inducing the dissemination of carcinoma [95, 96].

A ‘precancer niche’ is a prerequisite for the survival and development of EMT-initiated carcinoma cells [91]. Similarly, deriving from murine experimental models of metastasis, creation of a premetastatic niche at presumptive sites of metastasis is critical. It starts with a premetastatic inflammatory environment that will be seeded by micrometastatic circulating tumor cells [97].

**Fig. 3:**
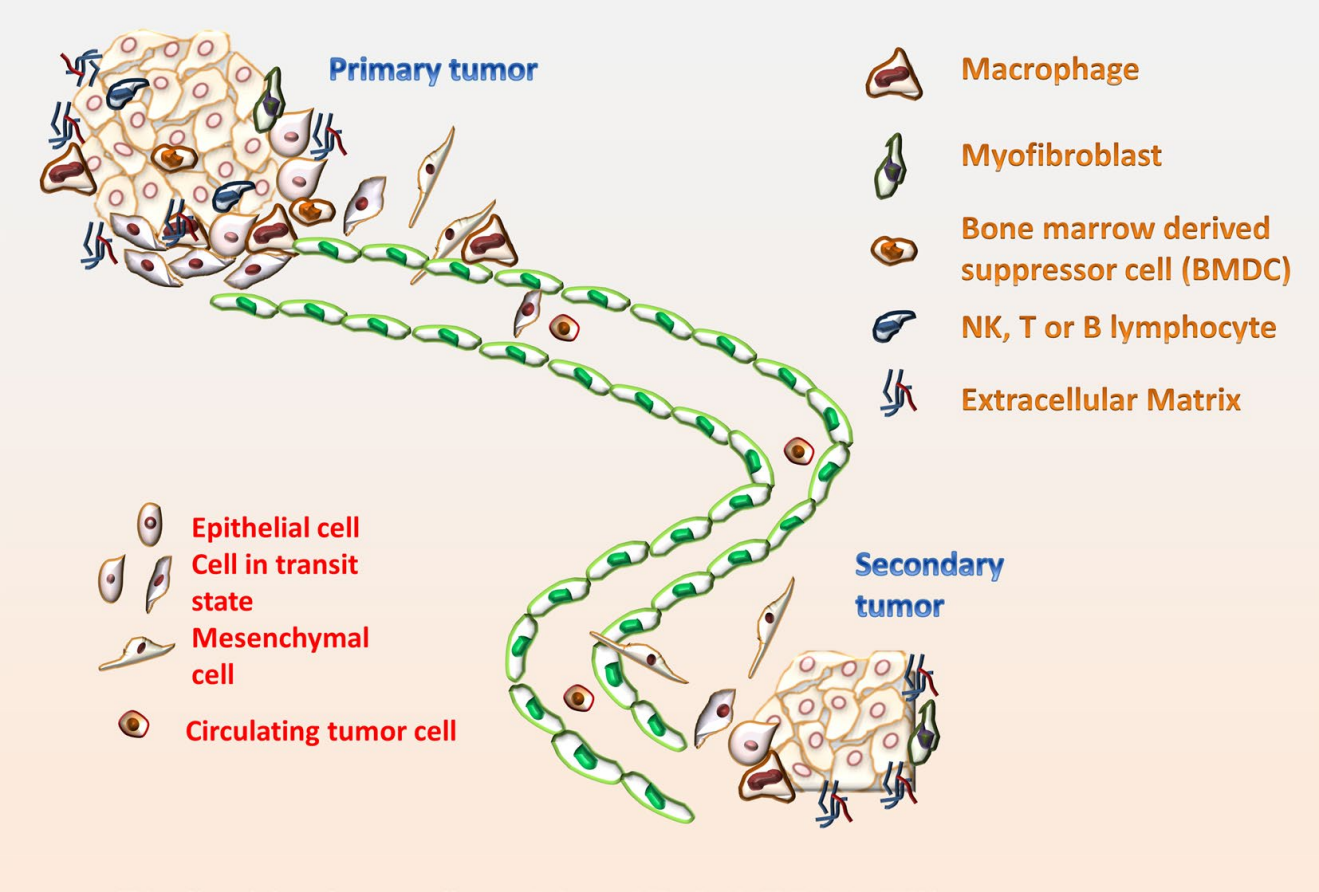
EMT in the metastatic cascade. Distinct stromal cells promote the dissemination of carcinoma cells, such as myofibroblasts, natural killer cells, T or B lymphocytes and macrophages. The scheme outlines the transition from an epithelial-like phenotype to a mesenchymal-like phenotype during local invasion and intravasation. Carcinoma cells can re-acquire through MET an epithelial-like phenotype following extravasation colonization of the secondary sites and proliferation. Macrophages can initiate a feedback-loop of EGF and Colony Stimulating Factor 1 (CSF1) to promote EMT. Myofibroblasts produce and reprogram ECM, secrete pro-inflammatory factors, and induce epithelial cell proliferation and invasion. BMDC contribute to the genesis of the premetastatic niche. Lymphocyte trafficking is also an important metastasis regulator. MDSCs interact with innate/adaptive immune cells, and promote cancer escape and angiogenesis in the cancer niche. MDSC and NK cells are also critical for MET process by populate premetastatic niches, where they regulate metastatic dissemination, initiate and promote tumor colonization

These circulating tumor cells (CTC) colonize and recruit endothelial progenitor cells (EPCs) that activate the angiogenic switch and shift micro-metastasis towards macro-metastasis [98]. Figure 3 describes the metastatic cascade in relation to the tumor stroma. EPCs are a subset of bone marrow-derived cells (BMDCs) that possess EndMT capabilities. BMDCs include macrophages, myeloid-derived suppressor cells (MDSCs), TIE2-expressing monocytes (TEMs), mesenchymal stem cells (MSCs), and platelets [34, 99]. Recent findings suggest exosomes from tumors regulate the immune response and hematopoietic system. These exosomes contain proteins and nucleic acids that induce lineage-specific differentiation of bone marrow precursors [91, 100, 101], and recruit inflammatory cells (such as MDSC) to distant sites [102].

Individual cell types within the tumor niche cells play distinct roles on tumor niche formation and cancer metastasis. Tumor-associated macrophages (TAMs) have a range of stromal cell types with significantly different physiological functions represented by polarized states M1, M2a, M2b and M2c [103]. It is hypothesized that TAMs exist either as a mixture of different polarized states or exhibit intermediary stages possessing partial functions of each polarized state [104, 105]. Macrophages can initiate a feedback loop of EGF and Colony Stimulating Factor 1 (CSF1) to promote EMT [106]. Most macrophages found in evolving tumors adopt the M2 polarized state, an immunosuppressive state-of-cell that is cancer promoting. Interestingly, different cell types within each family of macrophages and cytokines act upon specific cancers and sometimes, different regions of the same tumor. Proinflammatory M1-like TAMs are uniquely found in colorectal cancers and unlike M2 macrophages, suppress tumor progression by mediating cell killing [107]. Qingyihuaji Formula (QYHJ), a seven-herb patented TCM prescription, has significant anti-metastatic effects on pancreatic cancer even in gemcitabine resistant ones. QYHJ inhibits cancer-related inflammation in tumors by decreasing infiltration of tumor-associated macrophages and production of IL-6 [67].

MDSC is another cancer niche cell type and share the same progenitors as TAMs. They suppress immune responses to newly displayed tumor antigens. Thus, an increase in the heterogeneous collection of immature myeloid cells is observed in the bone marrow and blood of cancer patients [108]. MDSCs promote EMT possibly through TGF-β signaling, or via S100 series proteins [109]. Sheng Qi Formula (SQF) is commonly used to minimize chemotherapy side effects and as a complimentary cancer treatment. In vivo mouse model studies found that SQF formula significantly inhibited 4T1 mammary tumor development [81]. SQF treatment inhibited hydrogen peroxide production by Gr1^+^CD11b^+^ cells which in turn, reduced MDSCs protective effect on cancer cells. MDSCs interact with adaptive immune cells, to promote cancer escape and angiogenesis in the cancer niche [91, 110]. Feiyanning Decoction (FYN) is an antitumor prescription that restores T cell toxicity. In the Lewis lung carcinoma model, FYN was found to lower the ratio of CD4^+^CD25^+^ regulatory T cells (TREG) and down-regulate Foxp3 oncogene expression in TREG [80].

### Cancer EMT and chronic inflammation

The presence of chronic inflammation is strongly associated with EMT and the dynamic recruitment of BMDCs [50]. Inflammatory cytokine tumor necrosis factor (TNF-α), through NF-κB signaling, stabilizes SNAI1 for inflammation-induced metastasis [111]. Upon NF-κB p65 and IKK-β activation, EMT transcription factor TWIST1 expression is enhanced [112]. NF-κB signaling forms a positive feedback-loop which maintain high levels of pro-inflammatory cytokine (IL-1, TNF-α, IL-6), chemokine (IL-8, MIP-1, MCP-1), adhesion molecule (e.g. ICAM, VCAM) and growth factors [113]. As a result, inflammatory response cells like TAMs, T-cells and fibroblasts are recruited and accumulation of hypoxia inducible factor 1 (HIF-1) is favored [114]. Hypoxic responses induced by HIF-1 is also associated with activation of Twist1 and Snail1 [115]. Yi Ai Fang (YAF), a TCM formula, has been found to be effective against vasculogenic mimicry (VM) in colorectal cancer via HIF-1α/EMT regulation [116]. Another example is matrine, a natural alkaloid isolated from the traditional herb medicine sophora flavescens. This compound inhibited EMT in pancreatic cancer cells via ROS/NF-κB/MMPs pathway regulation [117].

## New “players” in EMT

EMT is a process which involves the dynamic cooperation of different hierarchical pathways involving multiple regulatory layers. Firstly, it is governed by transcriptional/translational changes in an orchestrated manner as discussed in previous paragraphs. Secondly, it is also regulated epigenetically and post-translationally. The activity of EMT transcription factors of the Snail, Zeb and Twist families is dependent on different epigenetic modifiers [79]. Epigenetic control of gene expression via DNA methylation on CpG islands is a conventional mechanism for silencing tumor suppression genes in tumor cells [118]. Epigenetic modification of histones is also involved in EMT where *SNAI1* induces repressive histone modifications through histone deacetylase (HDAC1) and HDAC2 [119]. Several other histone modifiers have been associated to *SNAI1* functions, such as demethylase LSD1, methyltransferase EZH2 and SUZ12 [62, 79, 120, 121].

MicroRNAs are also important in regulation of EMT inducers [122]. The family of miR-200 and the Zeb family of transcription factors control EMT and MET through negative feedback loops [123, 124]. *SNAI1*-dependent EMT is directly regulated by miR-34 family [125, 126]. Ginsenoside Rb1, extracted from the same plant as Ginsenoside Rb3, lowered miR-25 levels and inhibited cell migration abilities in SKOV3 and 3AO human ovarian cancer cells. The treatment reversed hypoxia-induced EMT by effecting on EP300 and E‑cadherin [66]. Genistein, a flavonoid found in a TCM herb Sophora japonica, promoted apoptosis while inhibiting prostate cancer cell proliferation, invasion and Wnt signaling in prostate cancer. MiR-1260b is highly expressed in prostate cancer tissues but genistein treatment significantly reduced it. sFRP1 and SMAD4 expression that are decreased in prostate cancer tissues can be partially restored by genistein via DNA demethylation and histone modifications [127].

Alternative splicing was recently reported to control EMT. Two RNA binding proteins, epithelial splicing regulatory protein 1 and 2 were recognized as critical promoters of FGFR2 splicing. The FGFR2b isoform is exclusively expressed in epithelial cell while the FGFR2c in mesenchymal cells [79, 128]. Jin-Fu-An decoction decreased the expression of p120ctn, isoform 1A, and its S288 phosphorylation, which reduced metastasis of H1650 lung cancer cell [129].

## Conclusions

In this review, the physiological and pathological EMT driven processes are summarized and the regulatory roles of TCM herbs and prescriptions on these processes are studied. The complexity of the EMT process demands efficient therapeutic interventions that have multiple layers of regulation. This criterion can be met by newly discovered or ancient TCM-derived therapies backed with modern evidence-based studies. Scientists found the treatments useful in reducing local invasion, distant dissemination, chemotherapy resistance, and cancer immune escape. Deep sequencing of cancer genomes has shown that stable driver mutations are difficult to identify in most solid tumors as they contain thousands of mutations on average and constantly evolve during tumor progression. Thus, multi-targeted therapies, such as TCM-derived therapies, regulating the unstable global status of cancer cells is significant in improving treatment response [18, 130, 131]. Thus, TCM is one strategy which can bring up new opportunities of generic treatment with the concept of evidence-based therapy.

Another challenge in cancer research remains in global mapping of EMT epigenetic modifications and related therapeutic opportunities. Technical developments in omics should provide better profiling analysis of translational interactions within cells in the EMT spectrum. TCM drugs have shown efficacy in targeting epigenetic modifications associated with EMT. However, the underlying mechanisms involved in its effect will require greater investigation. Current challenges remain such as isolating and identifying individual effective components from TCM, searching for lead compounds in herbal medicine, minimizing toxicity and establishing standard guidelines for clinical trials. Nonetheless TCM-derived therapies still hold great potential and the significant increase in studies to deconvolute heterogeneous herbal mixtures will aid modern evidence-based study of TCM on EMT and cancer research.

## Data Availability

All data included in this article are available from the corresponding author upon request.

## Abbreviations

AJ: adherens junction
BMDCs: bone marrow-derived cells
CTC: circulating tumor cells
CSF1: colony stimulating factor 1
COX2: cyclooxygenase2
EPCs: endothelial progenitor cells
EGF: epidermal growth factor
EMT: epithelial–mesenchymal transition
FGF: fibroblast growth factor
*FOXC2*: Forkhead boxC2
Gli1: glioma-associated oncogene homolog 1
HGF: hepatocyte growth factor
*HMGA2*: high mobility group A2
HIF-1: hypoxia inducible factor 1
IGF: insulin-like growth factor
LPS: lipopolysaccharides
MDCK: Madin–Darby canine kidney
MSCs: mesenchymal stem cells
MET: mesenchymal-to-epithelial transition
MMP: metalloprotease
MDSCs: myeloid-derived suppressor cells
*PRX1*: paired related homeobox 1
PDGF: platelet-derived growth factor
PEG2: prostaglandinE2
PKC: protein kinase C
RTK: receptor tyrosine kinases
TREG: regulatory T cells
3D: three-dimensional
TEMs: TIE2-expressing monocytes
TJ: tight junction
TCM: Traditional Chinese Medicine
TGF-β: transforming growth factor-beta
TNF-α: tumor necrosis factor
TAMs: tumor-associated macrophages
VM: vasculogenic mimicry
Wnt: wingless-type
*ZEB1* and *ZEB2*: zinc finger E-box binding homeobox 1 and 2
